# Multi-Parameter Optimization of Rubidium Laser Optically Pumped Magnetometers with Geomagnetic Field Intensity

**DOI:** 10.3390/s23218919

**Published:** 2023-11-02

**Authors:** Kun Xu, Xiuyan Ren, Yujie Xiang, Mingxu Zhang, Xiang Zhao, Kexin Ma, Yaqi Tian, Dan Wu, Ziqiang Zeng, Guobao Wang

**Affiliations:** Department of Nuclear Technology and Application, China Institute of Atomic Energy, Beijing 102413, China

**Keywords:** rubidium atomic gas cell, atomic magnetometer, magnetometer, sensitivity, magnetic field measurement

## Abstract

Rubidium laser optically pumped magnetometers (OPMs) are widely used magnetic sensors based on the Zeeman effect, laser pumping, and magnetic resonance principles. They measure the magnetic field by measuring the magnetic resonance signal passing through a rubidium atomic gas cell. The quality of the magnetic resonance signal is a necessary condition for a magnetometer to achieve high sensitivity. In this research, to obtain the best magnetic resonance signal of rubidium laser OPMs in the Earth’s magnetic field intensity, the experiment system of rubidium laser OPMs is built with a rubidium atomic gas cell as the core component. The linewidth and amplitude ratio (LAR) of magnetic resonance signals is utilized as the optimization objective function. The magnetic resonance signals of the magnetometer experiment system are experimentally measured for different laser frequencies, radio frequency (RF) intensities, laser powers, and atomic gas cell temperatures in a background magnetic field of 50,765 nT. The experimental results indicate that optimizing these parameters can reduce the LAR by one order of magnitude. This shows that the optimal parameter combination can effectively improve the sensitivity of the magnetometer. The sensitivity defined using the noise spectral density measured under optimal experimental parameters is 1.5 pT/Hz^1/2^@1 Hz. This work will provide key technical support for rubidium laser OPMs’ product development.

## 1. Introduction

High-precision weak-magnetic-field measurement instruments, magnetometers, have great potential for use in geological exploration [1], space exploration [2], magnetocardiography and magnetoencephalography measurement [3,4,5,6,7], nondestructive testing [8,9], nuclear magnetic resonance [10], and other fields [11]. According to the working principle, magnetometers can be divided into fluxgate magnetometers, nuclear spin resonance magnetometers (proton magnetometers, Euler Hauser effect proton magnetometers, and ^4^He nuclear spin magnetometers), electron precession magnetometers (Mz optically pumped magnetometers (OPMs), Mx OPMs, coherent population trapping (CPT), nonlinear magneto-optic rotating (NMOR), spin-exchange relaxation free (SERF)), and superconductor quantum interference devices (SQUIDs). Electron precession magnetometers do not require low-temperature cooling and have developed rapidly in recent years. In particular, the sensitivity of SERF all-optical electron precession magnetometers can reach the fT or even aT level in near-zero magnetic fields [12,13,14,15]. There is significant application potential in magnetocardiography and magnetoencephalography measurements. The sensitivity of OPMs is lower than that of a SERF magnetometer, but it has a large measurement range and can detect weak magnetic anomaly signals in a geomagnetic field. Therefore, it is a crucial magnetic field measurement instrument that has received widespread attention in recent years [16,17,18,19].

To improve the sensitivity and other performance indicators of OPMs that can be applied in a geomagnetic field, various new physical mechanisms and technologies have been applied to OPMs [16,19,20]. In recent years, with the development of laser technology, the application of lasers in OPMs has become a focus of attention owing to its good spectral line selectivity and stronger light intensity than spectral lamps. As a key instrument in the field of quantum measurement technology, laser OPMs have a sensitivity that is more than one order of magnitude higher than traditional light OPMs [21,22]. At present, the core materials available for OPMs include He, K, Rb, Cs, Na, and their mixed fillers [18,23,24,25,26]. Rubidium laser OPMs, which use high-abundance ^87^Rb isotopes as the core material, have advantages such as high sensitivity for magnetic anomaly detection, miniaturization, and no need for low-temperature conditions, making them an international research focus. The working mechanism of rubidium laser OPMs is to measure a magnetic field by measuring the intensity of the magnetic resonance signal passing through the rubidium atomic gas cell. The quality of the magnetic resonance signal is a necessary condition for a magnetometer to achieve high sensitivity. The experimental parameters of an OPM directly affect the LAR of the resonance signal.

Some researchers have optimized the experimental parameters that influence the sensitivity of cesium and helium OPMs applied in geomagnetic fields. For example, Shi et al. analyzed the influence of radio frequency (RF) power and the buffer gas pressure on the sensitivity of an optically pumped cesium magnetometer [27]. Zhang et al. analytically and experimentally explored suitable parameters based on frequency values and power densities of excitation to achieve optimal performances [28]. There has been optimization research on a single parameter for rubidium SERF magnetometers [29] and Mx magnetometers [24], which has reference significance for rubidium laser Mz OPMs. Nevertheless, the magnetic resonance signal of rubidium laser OPMs is affected by multiple working parameters. In this study, multi-parameter optimization is conducted on laser Mz OPMs and the impact of multi-parameter coupling on the magnetic resonance signal of rubidium laser Mz OPMs is analyzed.

A high abundance rubidium 87 isotope is used as the core material, and a narrow linewidth continuous laser pump and radio frequency magnetic field resonance method is adopted to measure the magnetic field by measuring the changes in laser intensity passing through the rubidium atomic gas cell. First, the principle of a laser atomic magnetometer based on rubidium 87 isotopes is studied. Second, a rubidium 87 isotope laser optically pumped magnetometer experimental system is built, including key components such as high-frequency demagnetized heating and saddle-shaped RF coils. After that, under a geomagnetic field intensity of 50,765 nT, magnetic resonance signals of different radio frequency intensities, the laser power, and the atomic gas cell temperature are experimentally measured. The LAR of the magnetic resonance signal are compared. As an optimization objective function, the influence of experimental parameters on the magnetic resonance signal of the magnetometer is analyzed, providing an important parameter basis for the development of a domestically produced high-sensitivity miniaturized laser-pumped rubidium atomic magnetometer.

## 2. Theoretical Analysis

### 2.1. Principle of a Rubidium Atomic Magnetometer

**Figure 1:**
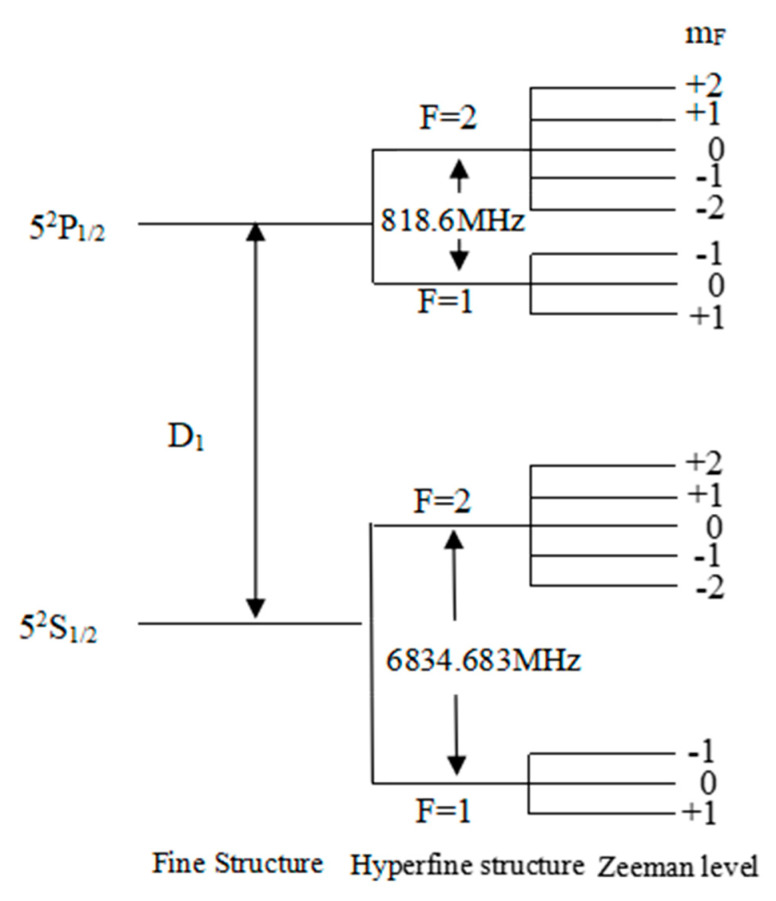
The atomic energy level structure of rubidium 87.

Owing to the coupling between the spin angular momentum of electrons and the orbital angular momentum of electrons, the values of the quantum number of the total angular momentum of electrons will be different, causing the energy levels of rubidium atoms to split, forming a fine structure of rubidium atoms. Because of the coupling between the total angular momentum of electrons and the nuclear spin angular momentum, the quantum number of the total angular momentum of atoms will have different values, causing further splitting of the energy levels of rubidium atoms, thus forming a hyperfine structure of rubidium atoms. Under the condition of an external magnetic field, the ground state energy level will undergo splitting, also known as Zeeman splitting. The degenerate hyperfine energy level of rubidium atoms will split into 2F + 1 Zeeman sublevels. Figure 1 illustrates the atomic energy level structure of rubidium 87.

In a rubidium atomic magnetometer, the energy of rubidium atoms in the ground state follows a Boltzmann distribution, and is generally uniformly distributed across the split energy levels of the ground state. The interval between adjacent Zeeman splitting energy levels is

(1) $$\Delta E=\hbar \omega =g_{s}\mu_{B}B,$$

where ℏ is the reduced Planck constant, ω is the Lamor precession frequency, $g_{s}$ is the Lande factor, $\mu_{B}$ is the Bohr magneton, and *B* is the measured magnetic field. According to Equation (1), the energy level interval is proportional to the size of the magnetic field, and measuring the energy level interval can determine the size of the magnetic field. In general, it is not convenient to directly measure ΔE, and the magnetic field can be measured by measuring the Lamor frequency ω.

**Figure 2:**
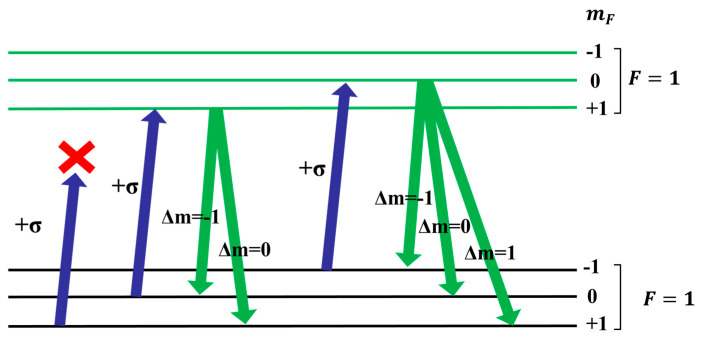
Rubidium atomic polarization process.

In a magnetic field, the ground state hyperfine structure of rubidium atom F = 1 splits into three sublevels, namely $m_{F}=-1,0,+1$. As Figure 2 shows, when the circularly polarized pump beam of the rubidium atom D1 line acts on the rubidium 87 atom, the atoms transition from ground state $F=1,m_{F}=-1,0$ levels to excited state $F=1,m_{F}=0,+1$ levels. Due to the absence of an excited state $F=1,m_{F}=+2$ energy level, atoms in the ground state $F=1,m_{F}=+1$ cannot absorb pump light. At the excited state energy level, the lifetime of an atom is very short, and the atom quickly falls back to the ground state $F=1,m_{F}=-1,0,+1$ through spontaneous emission. The atoms in the ground state $F=1,m_{F}=-1,0$ energy levels enter the pumping cycle again, while the number of atoms in the ground state $F=1,m_{F}=+1$ energy level increases. Ultimately, most atoms are in the ground state $F=1,m_{F}=+1$ splitting energy level. Atoms at this energy level are no longer able to absorb pump light, and the atoms reach a polarized state. At this point, the transmitted pump light intensity is the highest. Atoms in a polarized state can undergo transitions in an RF magnetic field, jumping to the adjacent Zeeman splitting energy level $F=1,m_{F}=0$. The polarization characteristics of atomic gases are disrupted, which is known as the depolarization phenomenon. At this point, atoms can absorb pump light again, and the intensity of transmitted light weakens.

When the frequency $\omega_{\mathrm{rf}}$ of the radio frequency magnetic field *B*_rf_ fulfills the following relationship, the polarization characteristics of atomic gases are most thoroughly disrupted, and the intensity of transmitted light is the lowest, reaching the state of magnetic resonance. From Equation (1), it can be concluded that

(2) $$B_{\mathrm{rf}}=\frac{\hbar \omega_{\mathrm{rf}}}{g_{s}\mu_{B}}=\frac{\omega_{\mathrm{rf}}}{\gamma_{\mathrm{Rb}}},$$

where $\gamma_{\mathrm{Rb}}$ is the spin magnetic ratio of rubidium 87 atoms, which is 6.99583 Hz/nT. Therefore, the magnetic field can be measured through resonance frequency $\omega_{\mathrm{rf}}$.

### 2.2. Bloch Equation of Laser Mz OPMs

To provide the influence of various experimental parameters on the magnetometer LAR, this article discusses the derivation of expressions for the linewidth and amplitude. First, in response to the work engineering of the magnetometer studied in this research, the Bloch equation of the macroscopic magnetic moment ***M*** with the combined influence of the measured external magnetic field *B*_0_ and the radio frequency magnetic field *B*_rf_ is obtained, taking into account the effects of relaxation and optical pumping. This is shown in the following Equation (3) [30]:

(3) $$\frac{d}{dt}\left(\begin{array}{c} M_{x}\\ M_{y}\\ M_{z} \end{array}\right)=\left(\begin{array}{c} M_{x}\\ M_{y}\\ M_{z} \end{array}\right)\times \left(\begin{array}{c} \gamma_{Rb}B_{rf}\cos\omega_{rf}t\\ \gamma_{Rb}B_{rf}\sin\omega_{rf}t\\ \gamma_{Rb}B_{0} \end{array}\right)-\left(\begin{array}{c} \gamma_{2}M_{x}\\ \gamma_{2}M_{y}\\ \gamma_{1}M_{z} \end{array}\right)+\Gamma_{P}\left(\begin{array}{c} M_{x}\\ M_{y}\\ M_{z}-M_{0} \end{array}\right),$$

where the macroscopic magnetic moment ***M*** has three components. *M*_0_ is the macroscopic magnetic moment at equilibrium. $\gamma_{1}$ is the longitudinal relaxation rate of atomic spin and $\gamma_{2}$ is the transverse relaxation rate of atomic spin. $\Gamma_{P}$ is the optical pumping rate.

At steady state, the above Equation (3) is zero. By solving the Bloch equation, the steady-state solution of *M*z is obtained as

(4) $$M_{Z}=\frac{\frac{\Gamma_{P}}{\Gamma_{P}-\gamma_{1}}[{\left(\gamma_{2}-\Gamma_{P}\right)}^{2}+{\left(\omega_{0}-\omega_{rf}\right)}^{2}]}{\omega^{2}\frac{\gamma_{2}-\Gamma_{P}}{\gamma_{1}-\Gamma_{P}}+{\left(\gamma_{2}-\Gamma_{P}\right)}^{2}+{\left(\omega_{0}-\omega_{rf}\right)}^{2}}M_{0}.$$

When the RF frequency $\omega_{rf}$ approaches $\omega_{0}$ and moves away from $\omega_{0}$, the maximum and minimum values of *M_z_* are obtained, and then the amplitude and half-width at half-height can be obtained, as shown in Equations (5) and (6). Δf is the linewidth of the magnetic resonance signal. ΔU is the amplitude of the magnetic resonance signal.

(5) $$\Delta f=\frac{\gamma_{2}-\Gamma_{P}}{\pi }\sqrt{1+\frac{\omega^{2}}{\left(\gamma_{1}-\Gamma_{P})(\gamma_{2}-\Gamma_{P}\right)}},$$

(6) $$\Delta U=\frac{\omega^{2}}{(\gamma_{1}-\Gamma_{P})(\gamma_{2}-\Gamma_{P})+\omega^{2}}\frac{\Gamma_{P}}{\Gamma_{P}-\gamma_{1}}M_{0}.$$

Equation (5) leads to the conclusion that the linewidth is primarily affected by relaxation and optical pumping. Both the numerator and denominator of Equation (5) contain $\gamma_{2}-\Gamma_{P}$. When $\Gamma_{P}$ approaches $\gamma_{2}$, the resonance signal will not appear. The magnetic moment is *M*_0_. But, theoretically, it is not possible to provide a linear or nonlinear expression for the relationship between linewidth and relaxation, and the same applies to optical pumping. Therefore, to obtain the optimal working parameters of the *M_z_* magnetometer, experiments are a good method that can truly reflect the influence of various parameters on the linewidth.

From Equation (6), it can be concluded that the amplitude of the magnetic resonance signal gradually increases with the decrease in the relaxation rate. The molecular denominator also has an optical pumping term, making it theoretically impossible to determine the effect of optical pumping on the amplitude of the magnetic resonance signal. The linewidth is mainly affected by relaxation.

The intrinsic sensitivity of a magnetometer is determined using LAR [31], which is the linewidth and amplitude of the magnetic resonance signal, as shown in Equation (7). To improve the intrinsic sensitivity of the magnetometer, it is required that the magnetic resonance signal has a smaller linewidth and a larger amplitude; that is, the smaller the ratio of the linewidth and amplitude, LAR, is, the higher the sensitivity of the magnetometer is.

(7) $$\mathrm{LAR}=\frac{\Delta f}{\Delta U}$$

## 3. Experiment Setup

**Figure 3:**
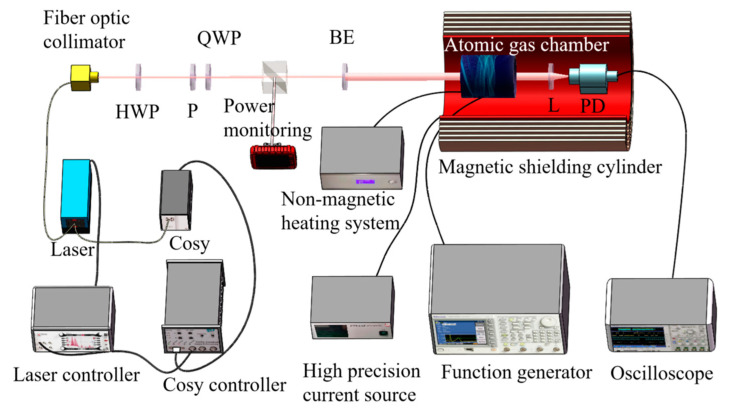
Schematic diagram of the experimental system for the Mz-type laser-pumped rubidium atomic magnetometer.

The experimental system of the laser-pumped rubidium atomic magnetometer mainly consists of a Toptica DLpro narrow linewidth external cavity semiconductor laser, laser frequency stabilization system (Cosy), Cosy controller, beam adjustment elements (half-wave plate (HWP), polarizer (P), quarter-wave plate (QWP), beam expander (BE), lens (L)), rubidium atomic gas cell, high-frequency demagnetized heating device, saddle-shaped RF coil, photodetector, torque-free coil, and magnetic shielding cylinder, as shown in Figure 3. A laser frequency stabilization system is used to lock the frequency generated with the laser, generating an ^87^Rb D1 line continuous monochromatic laser. A half-wave plate is used to adjust the optical power. A polarizer and quarter-wave plate form a circular polarizer, which is used to convert the laser generated with the laser into circularly polarized light. The beam expander is used to expand circularly polarized light into a spot with a diameter of approximately 15 mm, increasing the interaction area between the laser and rubidium atoms. The rubidium atomic gas cell is cylindrical in shape and has a size of Φ30 mm × 25 mm to reduce the collision of gaseous rubidium atoms on the inner wall of the gas cell after heating and increase the effective rubidium atoms that can be used for pump polarization. Two buffer gases, helium gas at 1000 Pa and nitrogen gas at 100 Pa, are used to fill the rubidium atomic gas cell. The photodetector (PD) utilizes a DSi200 ultraviolet-enhanced silicon detector. A moment-free coil is used to simulate the generation of a geomagnetic field, with the main magnetic field generated in the same direction as the laser. The rubidium 87 atomic gas cell is placed in a five-layer magnetic shielding cylinder with a residual magnetic field of 2 nT and used to shield the interference of external electromagnetic field signals with the experiment.

To facilitate the adjustment and monitoring of optical power, the beam adjustment components in the detection system are constructed using discrete components. The detection system, which is located inside the magnetic shielding cylinder, has other components integrated into it.

### 3.1. Magnetic Shielding System

Owing to the interference caused by different frequencies of magnetic field signals in the geomagnetic field and geomagnetic field in the laboratory environment, it is necessary to compensate for or shield the interference signals. Typically, active shielding (measuring interference to compensate for interference) or passive shielding (multi-layer magnetic shielding) methods are used for compensating for or shielding interference signals. To reduce magnetic interference signals and improve the convenience of experiments, a multi-layer magnetic shielding device is adopted to provide a nonmagnetic interference environment for conducting magnetic measurement experiments. The sensitivity testing and optimization of the magnetometer are carried out in the magnetic shielding device. The magnetic shielding device has two functions, the first of which is to shield the external magnetic field. In the geomagnetic environment, the residual magnetic field inside the barrel is required to be less than 2 nT. Furthermore, it can provide a shield for electromagnetic interference, and the magnetic shielding device contains a layer of an aluminum tube, which plays a role in shielding for electromagnetic interference.

The common shapes of magnetic shielding devices are spherical, cylindrical, and square. When using the same material, the spherical shielding factor is the highest, but the processing difficulty is the greatest and the spherical shape is inconvenient to use. The square shape is the easiest to machine, but due to its sharp edges, the shielding factor is the smallest. When the cylinder length is greater than three times the cylinder diameter, the shielding factor is close to that of the cylinder, and it is easy to process and use. Therefore, in general, a cylindrical structure is used. In this research, the magnetic shielding device is a cylindrical shielding cylinder, and the designed cylinder length is greater than three times the cylinder diameter. Usually, to increase shielding performance, a multi-layer structure is used. In this research, a six-layer structure is used with an outer layer of aluminum and a middle layer of five layers of a high-permeability permalloy (relative permeability is 2 × 10^4^–2 × 10^5^), with one end sealed and the other end covered, with a through hole in the center of both ends of Φ30 mm. The inner cavity size is Φ650 mm × 2500 mm.

**Figure 4:**
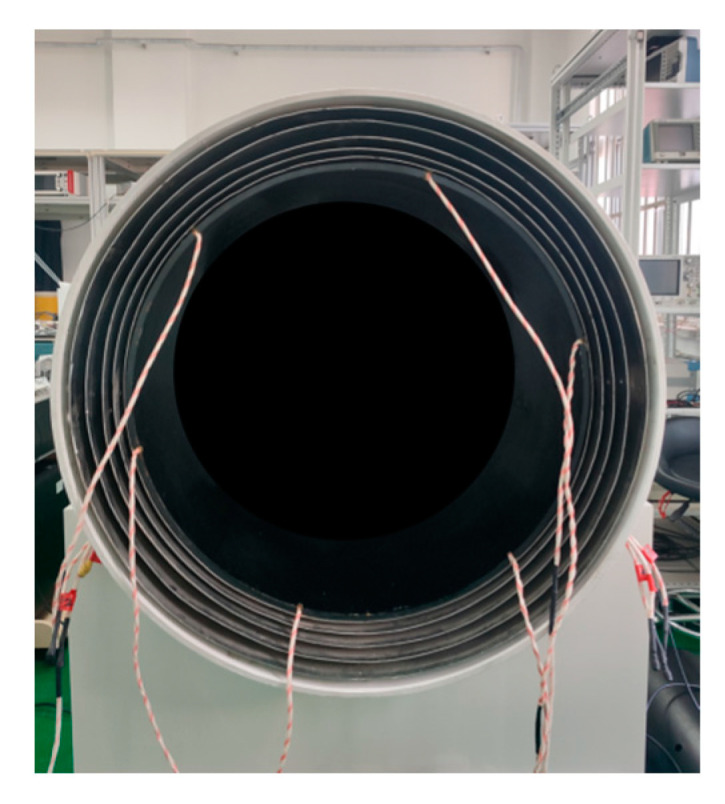
Magnetic shielding device.

The inner lining, cushion layer, and outer protective cover of the magnetic shielding device are designed with consideration of the actual usage environment. The inner surface is lined with nonmagnetic insulators, and the cushion layer meets the load-bearing requirements for placing torque-free coils in the later stage. The device is designed as a whole buckle cover, and one end is a movable cover structure that can be moved as a whole under the support of the support frame, ensuring a complete fit with the main body of the magnetic shielding device, as shown in Figure 4. To avoid magnetization of the magnetic shielding device after a period of use, a demagnetization line is designed for this device that is placed inside the shielding layer and equipped with a demagnetizer to achieve the purpose of demagnetizing the magnetic shielding device. After actual measurement, the magnetic shielding device is placed in the geomagnetic field environment in an axial east–west direction, with axial residual magnetic field B ≤ 2 nT and a radial horizontal residual magnetic field B ≤ 2 nT, meeting the requirements of the magnetometer magnetic measurement experiment.

### 3.2. High-Frequency Nonmagnetic Heating Device

**Figure 5:**
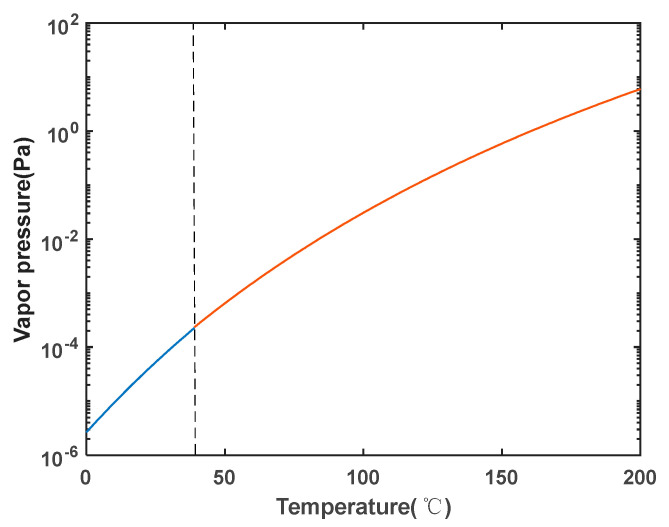
Vapor pressure curve of rubidium-87 isotope. (The blue curve applies to solid rubidium-87, while the red curve applies to liquid rubidium-87).

In the experiment, laser interaction with gaseous rubidium atoms is required, and the density of rubidium particles in the atomic cell is closely related to the temperature inside the cell. Typically, the density of rubidium particles in an atomic cell can be acquired using the saturation vapor pressure and the ideal gas state equation. This is based on the formulas for the saturation vapor pressure of solid rubidium-87 (Equation (8)) and liquid rubidium-87 (Equation (9)) [32]. The saturation vapor pressure curve for rubidium-87 is obtained, as shown in Figure 5. The melting point of rubidium is 38.89 °C. The particle density can be obtained using the ideal gas state equation (Equation (10)). Therefore, precise control of the temperature inside the atomic cell is crucial. Additionally, it is required that the heating device does not introduce any interfering magnetic fields.

(8) $$\log_{10}P=2.881+4.857-\frac{4215}{T},$$

(9) $$\log_{10}P=2.881+4.312-\frac{4040}{T},$$

(10) $$n=N_{A}\frac{P}{RT}.$$

For the atomic cell, conventional heating methods include DC coil heating, thin film heating, semiconductor heating, and high-temperature gas heating. However, common DC coil heating, thin film heating, and semiconductor heating methods all experience significant magnetic field interference issues. While high-temperature gas heating has very low magnetic noise, it is difficult to control the temperature precisely, and portability is lacking. Traditional open-loop DC coil heating methods have difficulty controlling the heating temperature and stabilizing it at the desired value. Moreover, they introduce significant magnetic field interference.

The high-frequency nonmagnetic heating system developed in this study consists of three components: the control system, heating module, and temperature sensing probe. The main function of the control system is to use a high-frequency current for heating, with a frequency of up to 10 kHz, avoiding the rubidium atomic magnetometer’s Larmor precession frequency. The interference magnetic field signals generated with the high-frequency current are filtered out using the backend circuitry. The heating module employs a twisted pair wire model where the metal wire is wound around the outside of the atomic cell heating cavity, allowing the magnetic fields generated with the current in the wire to cancel each other out in opposite directions. The temperature sensing probe uses platinum resistance Pt100. This heating and temperature sensing module also makes it possible to replace the cylindrical rubidium atomic cell in the experiment, thus eliminating the need for repetitive and irregular manual winding of heating coils. Finally, at 70 °C, the temperature control accuracy of the high-frequency nonmagnetic heating system is measured to be better than ±1.5 °C within 1 min.

### 3.3. Saddle-Shaped RF Coil

**Figure 6:**
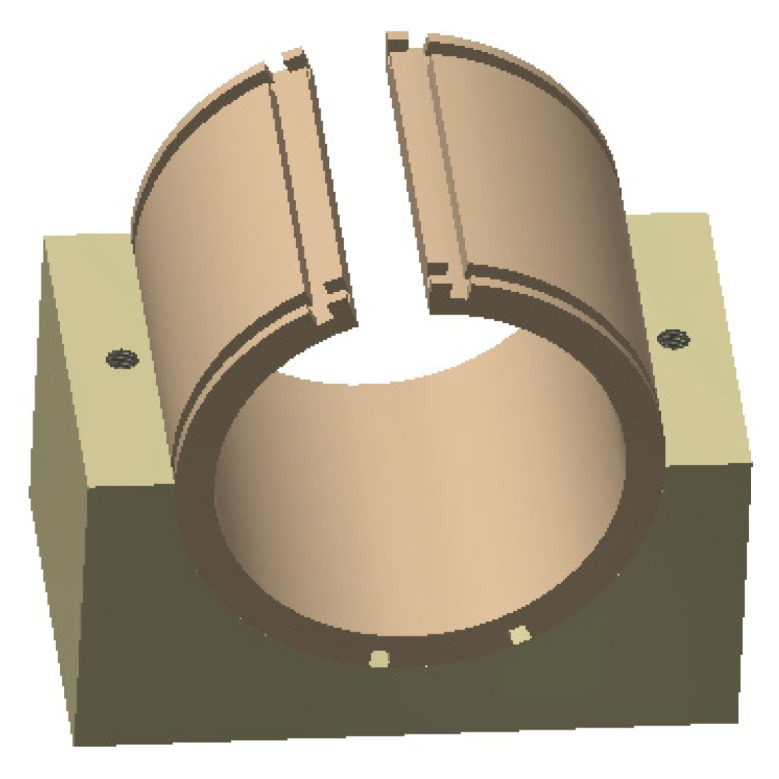
Saddle-shaped RF coil holder.

RF coils for magnetometers are generally designed as Helmholtz coils. However, Helmholtz coils are massive and inconvenient for installation and integration, especially when used with cylindrical Rubidium atomic cells. Hence, in this study, the RF coil is designed in a saddle shape to match the structure of the cylindrical Rubidium cell and the heating probe. Figure 6 illustrates the designed RF coil and its support bracket for the cylindrical Rubidium cell. The RF coil is wound with a fine copper wire along the grooves on both sides of the cylindrical support bracket, with consistent winding direction on both sides. The size of the RF coil bracket is Φ60 mm × 46 mm, and the RF coil is installed in the groove of the RF coil. The two coils are symmetrical, and each coil has four turns. The coil constant is about 100 nT/mA through finite element simulation. In the experiment, the saddle-shaped RF coil and its support bracket are placed on a semi-circular support stand. This compact RF coil design facilitates integration with the temperature control system and heating module, enabling miniaturization of the probe.

## 4. Results and Discussion

**Figure 7:**
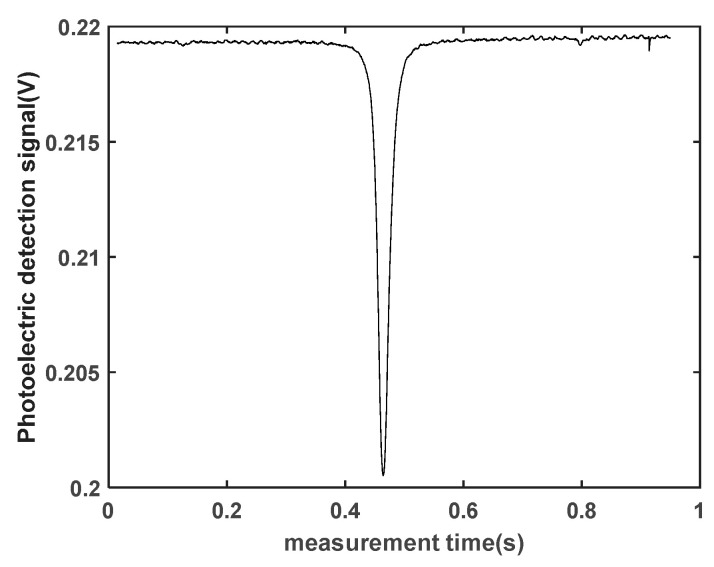
Magnetic resonance signal measured in the experiment.

In the magnetic field measurement experiment, the magnetic field being tested is generated with a nonmagnetic coil. The current is set to 1680 mA, producing a magnetic field of 50,765 nT. First, the temperature of the atomic vapor cell is adjusted using a temperature controller to convert the ^87^Rb isotope into a gaseous state using the nonmagnetic heating device. Then, the coupling power of the laser fiber coupling head is maximized with adjustment, and the laser frequency locking system is controlled through software to lock the laser frequency to the D1 transition spectrum of ^87^Rb. A monochromatic laser beam resonant with the D1 transition of ^87^Rb is used as the pump light, which is converted into circularly polarized light via a circular polarizer. The circularly polarized light enters the Rubidium atomic cell after being expanded with a beam expander, polarizing the ^87^Rb atomic gas. The polarized ^87^Rb atomic gas no longer absorbs the pump light, and the strongest light intensity detected with the photodetector is observed on the oscilloscope. Finally, the RF coil is connected to a function generator, which generates a sinusoidal frequency sweep signal. Given the magnetic field strength of 50,765 nT, the Larmor frequency is 355 kHz. Therefore, the frequency sweep range is set to 300–400 kHz, with a sweep time of 1 s. The intensity of the RF signal can be adjusted. When the RF magnetic field frequency matches the spin Larmor precession frequency of the atoms, the rubidium atoms are depolarized, and the weakest light intensity is observed on the oscilloscope. Figure 7 illustrates the magnetic resonance signal collected through the oscilloscope. The lowest point of the signal represents the magnetic resonance point, the half-width of the signal represents the linewidth, and the difference between the maximum and minimum values of the signal represents the signal amplitude. The frequency corresponding to the magnetic resonance point is the Larmor resonance frequency $\omega_{0}$, which can be calculated from Equation (2) to obtain the magnetic field value. 

### 4.1. Effect of the RF Intensity on the Magnetic Resonance Signal

**Figure 8:**
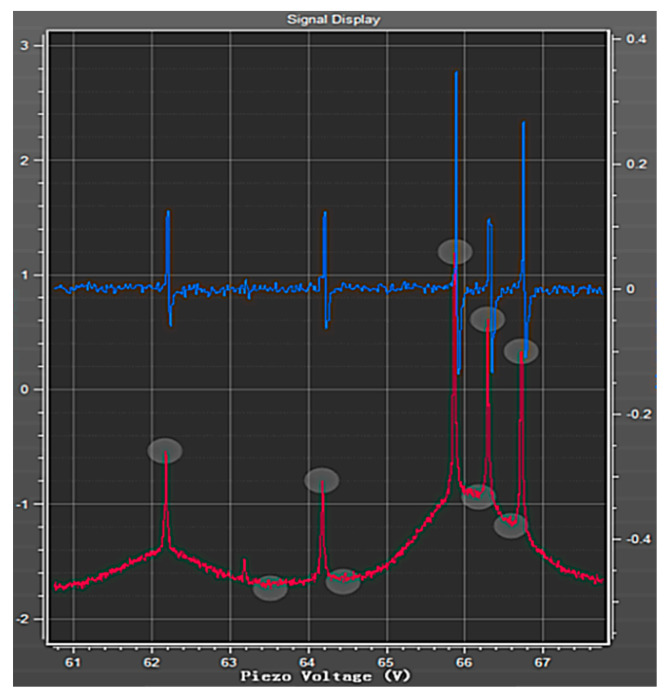
D1 absorption line of rubidium 87 atom (the blue curve) and rubidium 85 atom (the red curve).

From the mechanism of relaxation, it can be inferred that the change in the pump laser frequency affects the rate of relaxation, thereby affecting the linewidth. In Figure 8, the D1 absorption spectral lines of rubidium 87 and rubidium 85 atoms are shown. Based on the spacing between the ground state hyperfine energy levels of rubidium 87, the frequency difference between the first and third spectral lines is 814.5 MHz. In accordance with the spacing between the ground state hyperfine energy levels of rubidium 85, the frequency difference between the first and third spectral lines is 361.6 MHz. On the left side of the figure are three spectral lines of rubidium 87 from the ground state to the excited state: F = 2 to F = 1; F = 2 to F = 1 and F = 2; F = 2 to F = 2.

**Figure 9:**
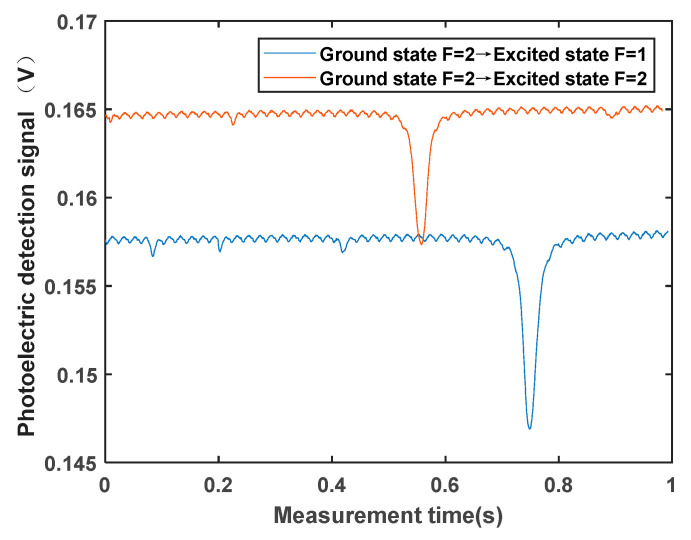
Effect of laser frequency on the sensitivity of magnetometers.

As shown in Figure 9, the effect of the laser frequency on the sensitivity of the magnetometer is given by selecting different absorption spectral lines to lock in the laser frequency. The first spectral line (ground state F = 2 to excited state F = 1) with the strongest signal strength and the smallest linewidth is selected. The third spectral line (ground state F = 2 to excited state F = 2) with a signal strength smaller than the first spectral line is also selected. Due to the low signal amplitude of the second spectral line ground state F = 2 to excited state F = 1 and excited state F = 2, it is almost impossible to capture its frequency point, and the signal amplitude is zero. Therefore, to improve the experimental sensitivity, the laser frequency is locked at the first spectral line in the subsequent experiments.

### 4.2. Effect of the RF Intensity on the Magnetic Resonance Signal

**Figure 10:**
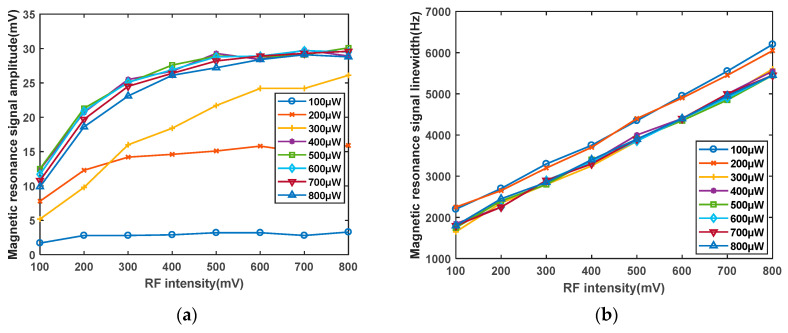
(**a**) Effect curve of RF intensity on magnetic resonance signal amplitude. (**b**) Influence curve of RF intensity on magnetic resonance signal linewidth.

**Figure 11:**
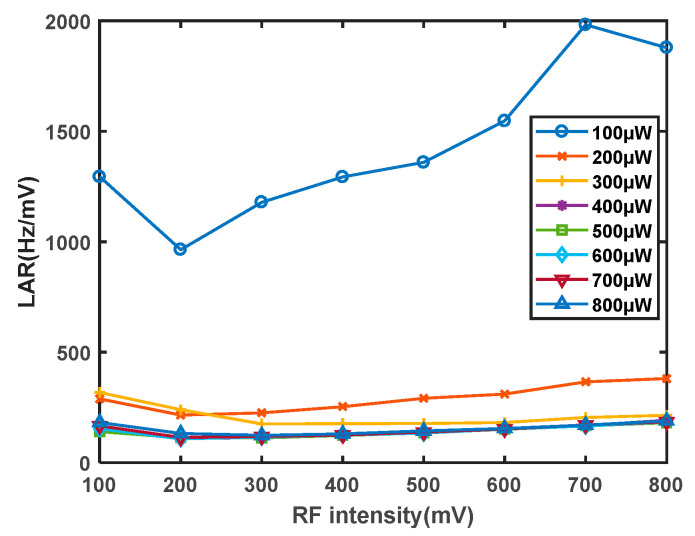
Influence curve of RF intensity on the ratio of magnetic resonance signal linewidth to amplitude.

First, The temperature of the rubidium atom cell is determined to be 70 °C. The laser frequency is set to 100 μW to 800 μW. The influence of the scanning field intensity of the RF coil on the magnetic resonance signal is studied. The RF signal cable is directly connected to the signal generator. The signal generator provides voltage signals to both ends of the coil. A current loop is formed in a closed coil, which in turn generates an alternating magnetic field. During the experiments, it is observed that when the radiofrequency intensity exceeds 800 mV, the signal amplitude does not show significant enhancement, but the linewidth increases notably. Therefore, the sweep field intensity of the radiofrequency coil is only varied within the range of 100 mV to 800 mV to investigate its effect on the sensitivity of the magnetometer. As shown in Figure 10a, the experimental findings reveal that as the radiofrequency intensity increases, the signal amplitude also increases, albeit at a decreasing rate. This phenomenon is attributed to the fact that polarized atoms require energy from the radiofrequency magnetic field for depolarization. When the radiofrequency intensity reaches a certain threshold, a saturation point is reached where a certain number of atoms have already undergone depolarization, leading to a gradual reduction in the rate of increase of the magnetic resonance signal. As shown in Figure 10b, the linewidth exhibits a nearly linear increase, with a slope greater than that of the signal amplitude increase. Simultaneously, the influence of the radiofrequency intensity on both the amplitude and linewidth is studied for different optical powers, showing similar trends. As depicted in Figure 11, after calculating the LAR of the amplitude to linewidth for different optical powers and radiofrequency intensities, it is observed that the impact of the radiofrequency intensity on the LAR is within the same order of magnitude and relatively minor. However, to achieve the optimal signal, the radiofrequency intensity should be maintained within the range of 200–300 mV.

### 4.3. Effect of Laser Power on the Magnetic Resonance Signal

**Figure 12:**
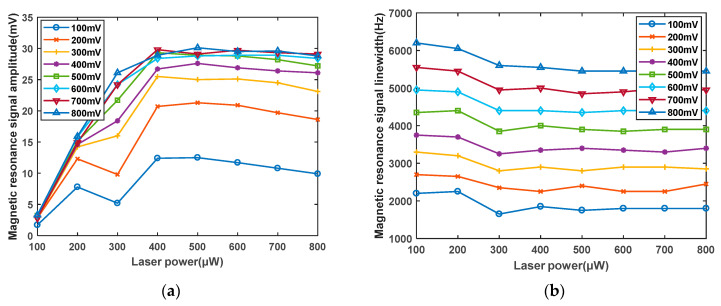
(**a**) Effect curve of optical power on magnetic resonance signal amplitude. (**b**) Influence curve of optical power on magnetic resonance signal linewidth.

**Figure 13:**
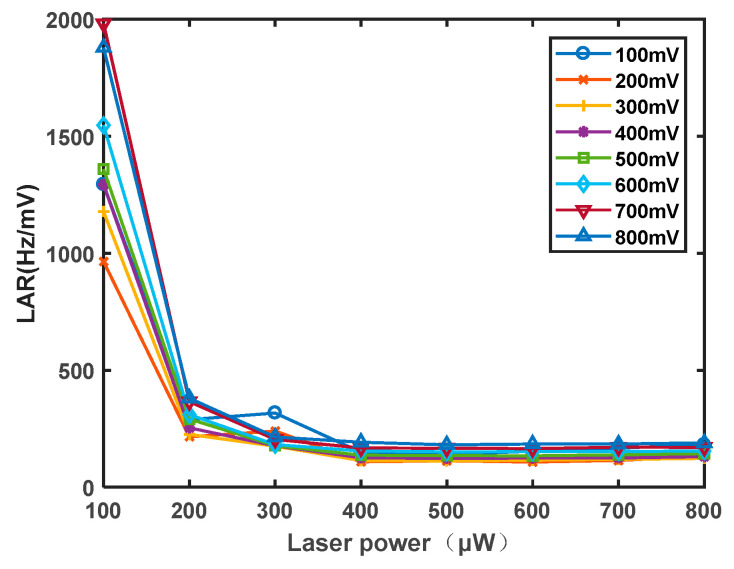
Influence curve of optical power on the ratio of magnetic resonance signal linewidth to amplitude.

After determining the range of radiofrequency intensity, in this study, the relationship between the laser power and the magnetic resonance signal is examined. As shown in Figure 12a, as the laser power increases, the signal amplitude gradually increases, although at a diminishing rate. This is because the pump laser can polarize ground-state atoms. As laser power increases, the polarization rate surpasses the relaxation rate, and when nearly all atoms are polarized, the rate of increase in the signal amplitude slows down and approaches saturation. As depicted in Figure 12b, the linewidth shows little variation with changes in the laser power, and relative to the signal amplitude, the influence of laser power on the linewidth is not significant. The impact of laser power on the amplitude and linewidth exhibits the same trends for different radiofrequency intensities. Figure 13 provides the curves illustrating the effect of the optical power on the magnetic resonance signal amplitude and linewidth. At 100 μW, the LAR is approximately 2000 Hz/mV, and at 400 μW, the LAR is approximately 200 Hz/mV. Adjusting the optical power can reduce the LAR ratio of the linewidth to amplitude by one order of magnitude. The optimal optical power has a minimum value, which should be greater than or equal to 400 μW. At this point, the ratio of the linewidth to amplitude, LAR, is the minimum.

However, the number of gaseous rubidium atoms present in the atomic vapor cell can vary at different temperatures, and consequently, the required laser power may not remain constant. In different temperature conditions, laser power cannot be held constant; it needs to be adjusted based on the number of rubidium atoms inside the vapor cell. When the atomic vapor cell temperature is high, there are more atoms, and higher laser power is required to polarize the atoms. Conversely, lower laser power is needed when there are fewer atoms. Therefore, in this study, systematic experimental research is conducted on the signal amplitude and linewidth under different temperature and laser power conditions.

### 4.4. Effect of the Atomic Cell Temperature on the Magnetic Resonance Signal

**Figure 14:**
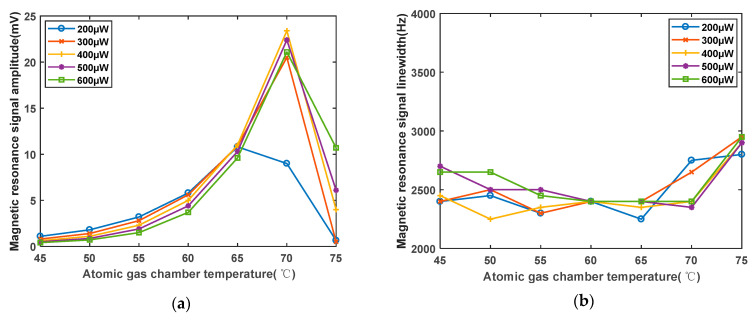
(**a**) Effect curve of atomic vapor cell temperature on magnetic resonance signal amplitude. (**b**) Influence curve of atomic vapor cell temperature on magnetic resonance signal linewidth.

The RF intensity is determined to be 200 mV, and the temperature and laser power of the high-frequency nonmagnetic heating system change. The temperature range is 45 °C to 75 °C. As shown in Figure 14a, experimental findings reveal that as the temperature of the atomic vapor cell increases, the signal amplitude initially increases and then decreases. This is attributed to the fact that as the temperature of the atomic vapor cell increases, the number of gaseous rubidium atoms available for pumping increases, leading to greater polarization. However, as the rubidium content within the atomic vapor cell is limited, the further temperature increase due to collision effects causes the effective number of gaseous rubidium atoms available for pumping to decrease rapidly, resulting in a reduction in the amplitude of the magnetic resonance signal. As shown in Figure 14b, the linewidth exhibits a trend of initially remaining stable and then gradually increasing. For laser powers of 200 μW and 300 μW, the linewidth begins to increase from 65 °C. For laser powers of 400 μW, 500 μW, and 600 μW, the linewidth begins to increase from 70 °C. However, for different laser powers, the optimal temperature points are different. At lower laser powers (200 μW), the optimal atomic vapor cell temperature is lower at 65 °C. When the power reaches 400 μW, the optimal temperature is 70 °C.

**Figure 15:**
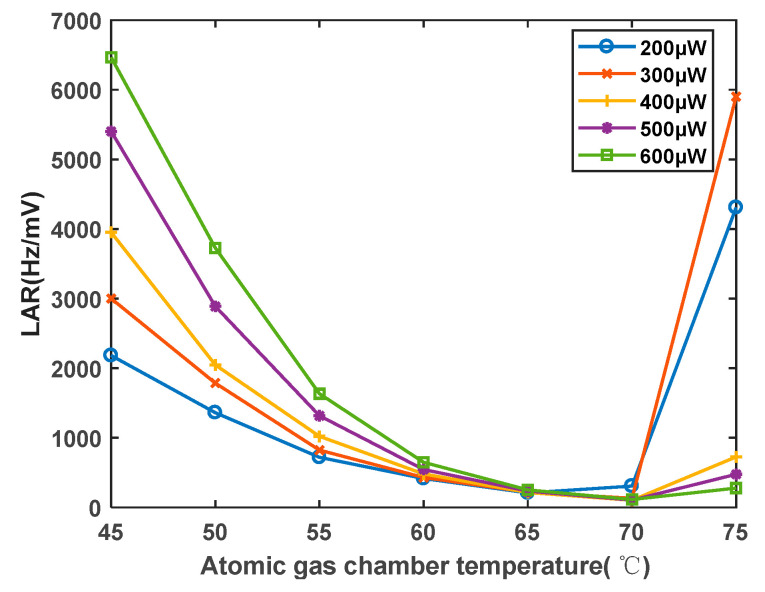
Influence curve of atomic vapor cell temperature on the ratio of magnetic resonance signal linewidth to amplitude.

Under varying laser power and temperature conditions, the ratio of the linewidth to amplitude (LAR) for the magnetic resonance signal exhibits the changes shown in Figure 15. The optimal combination of laser power and temperature is found to be 400 μW and 70 °C. At 45 °C, the LAR is approximately 4000 Hz/mV, while at 70 °C, the LAR is approximately 100 Hz/mV. The LAR decreased to 1/40 of its value before optimization, indicating that the sensitivity of the magnetometer can be improved by a factor of 40 after optimization. This provides a parameter-based foundation for enhancing magnetometer sensitivity.

The above optimization process has not yet considered the effects of the buffer gas type, pressure, and nonuniformity of the measured magnetic field on the linewidth. However, this research provides an experimental basis for optimizing the linewidth of other parameters for the same atomic vapor cell and external magnetic field. After replacing different atomic vapor cells, the best parameter combination for the atomic vapor cell can be quickly found according to the methods and rules described in this paper.

### 4.5. Measurement of Magnetometers’ Sensitivity under Optimal Experimental Parameters

**Figure 16:**
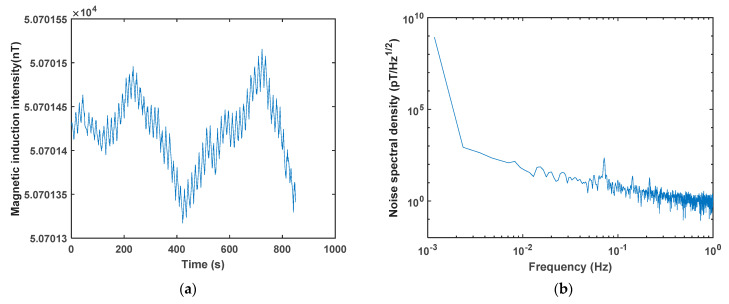
(**a**) The real-time measurement curve of magnetic induction intensity under optimal experimental parameters. (**b**) The noise spectral density measured under optimal experimental parameters.

The sensitivity of OPMs is an important indicator. The sensitivity mentioned in section two of this research is the inherent sensitivity of the magnetometer, which is applicable to the evaluation of parameter selection in open-loop systems. In order to obtain the measured sensitivity of the magnetometer under the optimized parameters in this research, we designed a closed-loop control system for this magnetometer based on LabVIEW and the data acquisition module, which can obtain the magnetic induction intensity of the measured magnetic field in real time. The measured sensitivity is characterized by noise spectral density. The real-time measurement curve of magnetic induction intensity obtained is shown in Figure 16a, with a peak-to-peak value of approximately 0.2 nT after about 15 min of real-time measurement. As shown in Figure 16b, the noise spectral density measured under optimal experimental parameters is 1.5 pT/Hz^1/2^@1 Hz. 

## 5. Conclusions

In this research, the magnetic measurement process of rubidium laser OPMs is analyzed, and the experimental system of rubidium laser OPMs is built. For the simulated geomagnetic field intensity, the magnetic measurement experiment of the magnetometer probe is carried out using the experimental system. The following conclusions are obtained:

(1) The observed effects of the radiofrequency (RF) intensity, laser power, and atomic cell temperature on the magnetic resonance signal amplitude, linewidth, and linewidth-to-amplitude ratio, LAR, are consistent with the theoretical analysis.

(2) The radiofrequency (RF) intensity has a comparatively minor impact on the linewidth-to-amplitude ratio, LAR, but should be controlled within the range of 200–300 mV. Laser power and atomic cell temperature are the primary factors influencing the amplitude, linewidth, and linewidth-to-amplitude ratio, LAR, of the magnetic resonance signal. Adjusting the laser power can reduce the linewidth-to-amplitude ratio, LAR, by one order of magnitude while altering the atomic cell temperature can reduce LAR to 1/40 of its value before optimization.

(3) At different atomic cell temperatures, the optimal laser power varies. Laser power should be adjusted based on the number of rubidium atoms inside the atomic cell. An optimal combination of laser power and atomic cell temperature can help achieve the best performance.

(4) The peak-to-peak value of magnetic induction intensity is approximately 0.2 nT after about 15 min of real-time measurement under optimal experimental parameters. The sensitivity defined using the noise spectral density measured under optimal experimental parameters is 1.5 pT/Hz^1/2^@1 Hz.

This article provides the optimal parameter selection reference for the development of highly sensitive Mz rubidium laser OPMs for use in a geomagnetic field. The next step will be to design an integrated miniaturized magnetic field sensing probe and a closed-loop lock-in circuit for the Larmor frequency based on the optimized experimental parameters, facilitating closed-loop magnetic field measurements and enhancing the real-time measurement capabilities of the magnetometer.

## Data Availability

Not applicable.

## References

[1] Bertrand F., Jager T., Boness A., Fourcault W., Le Gal G., Palacios-Laloy A., Paulet J., Léger J.M. (2021). A He-4 vector zero-field optically pumped magnetometer operated in the Earth-field. Rev. Sci. Instrum..

[2] Korth H., Strohbehn K., Tejada F., Andreou A.G., Kitching J., Knappe S., Lehtonen S.J., London S.M., Kafel M. (2016). Miniature atomic scalar magnetometer for space based on the rubidium isotope Rb-87. J. Geophys. Res. Space Phys..

[3] Mooney J.W., Ghasemi-Roudsari S., Banham E.R., Symonds C., Pawlowski N., Varcoe B.T.H. (2017). A Portable Diagnostic Device for Cardiac Magnetic Field Mapping. Biomed. Phys. Eng. Express.

[4] Strand S., Lutter W., Strasburger J.F., Shah V., Baffa O., Wakai R.T. (2019). Low-Cost Fetal Magnetocardiography: A Comparison of Superconducting Quantum Interference Device and Optically Pumped Magnetometers. J. Am. Heart Assoc. Cardiovasc. Cerebrovasc. Dis..

[5] Tierney T.M., Holmes N., Mellor S., López J.D., Roberts G., Hill R.M., Boto E., Leggett J., Shah V., Brookes M.J. (2019). Optically pumped magnetometers: From quantum origins to multi-channel magnetoencephalography. NeuroImage.

[6] Yang Y., Xu M., Liang A., Yin Y., Ma X., Gao Y., Ning X. (2021). A new wearable multichannel magnetocardiogram system with a SERF atomic magnetometer array. Sci. Rep..

[7] Zhang R., Xiao W., Ding Y., Feng Y., Peng X., Shen L., Sun C., Wu T., Wu Y., Yang Y. (2020). Recording brain activities in unshielded Earth’s field with optically pumped atomic magnetometers. Sci. Adv..

[8] Bevington P., Gartman R., Chalupczak W., Deans C., Marmugi L., Renzoni F. (2018). Non-destructive structural imaging of steelwork with atomic magnetometers. Appl. Phys. Lett..

[9] Bevington P., Gartman R., Chalupczak W. (2019). Alkali-metal spin maser for non-destructive tests. Appl. Phys. Lett..

[10] Oida T., Tsuchida M., Kobayashi T. (2012). Direct Detection of Magnetic Resonance Signals in Ultra-Low Field MRI Using Optically Pumped Atomic Magnetometer With Ferrite Shields: Magnetic Field Analysis and Simulation Studies. IEEE Trans. Magn..

[11] Ruan Y., He X., Ruan L.M., Liu F., Zhang Z., Weng J., Yan Q., Zhang G., Li K., Zheng W. (2022). Drug Monitoring by Optically Pumped Atomic Magnetometer. IEEE Photonics J..

[12] Kominis I.K., Kornack T.W., Allred J.C., Romalis M.V. (2003). A subfemtotesla multichannel atomic magnetometer. Nature.

[13] Fu Y., Wang Z., Xing L., Fan W., Ruan J., Pang H. (2022). Suppression of Nonuniform Magnetic Fields in Magnetic Shielding System for SERF Co-Magnetometer. IEEE Trans. Instrum. Meas..

[14] Guo Q., Li X.W., Zhang N., Lu J., Ma D., Li Z., Jiang S. (2022). The Space Density Distribution of Alkali Metal Atoms in a SERF Atomic Magnetometer. IEEE Sens. J..

[15] Liang Z.H., Zhou B.Q., Lu J.X., Liu Y., Hu J., Zhou P., Wang W., Liu L., Hu G., Ye M. (2022). Metasurface enabled on-chip double-beam scheme for SERF atomic magnetometer. Opt. Commun..

[16] Oelsner G., Schultze V., Ijsselsteijn R., Stolz R. (2019). Performance analysis of an optically pumped magnetometer in Earth’s magnetic field. EPJ Quantum Technol..

[17] Liu L., Lu Y., Zhuang X., Zhang Q., Fang G. (2020). Noise Analysis in Pre-Amplifier Circuits Associated to Highly Sensitive Optically-Pumped Magnetometers for Geomagnetic Applications. Appl. Sci..

[18] Wang H., Wu T., Wang H., Li S., Lin Z., Peng X., Guo H. (2020). A Compact Laser Pumped 4He Magnetometer with Laser-Frequency Stabilization by Inhomogeneous Light Shifts. Appl. Sci..

[19] Oelsner G., Ijsselsteijn R., Scholtes T., Krüger A., Schultze V., Seyffert G., Werner G., Jäger M., Chwala A., Stolz R. (2022). Integrated Optically Pumped Magnetometer for Measurements within Earth’s Magnetic Field. Phys. Rev. Appl..

[20] Zhang L.-L., Bai L.-L., Yang Y.-L., Yang Y.-B., Wang Y.-H., Wen X., He J., Wang J.-M. (2021). Improving the sensitivity of an optically pumped rubidium atomic magnetometer by using of a repumping laser beam. Acta Phys. Sin..

[21] Groeger S., Pazgalev A.S., Weis A. (2005). Comparison of discharge lamp and laser pumped cesium magnetometers. Appl. Phys. B.

[22] Wang C., Zhou Z., Cheng D. (2018). Research on the signal of 4 He pump magnetometer sensor using ECDL laser. Biomed. Res..

[23] Zhang X.-X., Zheng H.-Q., Ge L.-L., Zhao H., Ren Q.-Y., Ma X.-Q. (2020). Theoretical and experimental results of optically pumped sodium atoms. Opt. Commun..

[24] Ding Z.C., Yuan J., Wang Z., Lu G., Luo H. (2016). Optically pumped rubidium atomic magnetometer with elliptically polarized light. Optik.

[25] Groeger S., Bison G., Weis A. (2005). Design and performance of laser-pumped Cs-magnetometers for the planned UCN EDM experiment at PSI. J. Res. Natl. Inst. Stand. Technol..

[26] Fang J., Wang T., Quan W., Yuan H., Zhang H., Li Y., Zou S. (2014). In situ magnetic compensation for potassium spin-exchange relaxation-free magnetometer considering probe beam pumping effect. Rev. Sci. Instrum..

[27] Shi R.-Y., Wang Y.-H. (2013). Analysis of influence of RF power and buffer gas pressure on sensitivity of optically pumped cesium magnetometer. Chin. Phys. B.

[28] Zhang J., Wang Y.Z., Wang C., Zhou Z., Li W. (2022). Performance Enhancement by Investigating on Excitation Parameters of Helium Cell in He-4 Optically Pumped Magnetometer. IEEE Sens. J..

[29] Chen C., Liu X., Qu T., Yang K. Optimization of buffer gas pressure for Rb atomic magnetometer. Proceedings of the 2015 International Conference on Optical Instruments and Technology: Optical Sensors and Applications.

[30] Bloch F. (1946). Nuclear Induction. Phys. Rev..

[31] Kawabata R., Fukuda K., Kandori A. (2010). Optimized Condition for Buffer Gas in Optical-Pumped Magnetometer Operated at Room Temperature. Jpn. J. Appl. Phys..

[32] Alcock C.B., Itkin V.P., Horrigan K. (1984). Vapour Pressure Equations for the Metallic Elements: 298–2500K. Can. Metall. Q..

